# Association of Online Search Trends With Vaccination in the United States: June 2020 Through May 2021

**DOI:** 10.3389/fimmu.2022.884211

**Published:** 2022-04-20

**Authors:** Philipp Berning, Leu Huang, Alexander C. Razavi, Ellen Boakye, Ngozi Osuji, Andrew C. Stokes, Seth S. Martin, John W. Ayers, Michael J. Blaha, Omar Dzaye

**Affiliations:** ^1^ Johns Hopkins Ciccarone Center for the Prevention of Cardiovascular Disease, Johns Hopkins University School of Medicine, Baltimore, MD, United States; ^2^ Department of Hematology and Oncology, University Hospital Muenster, Muenster, Germany; ^3^ Department of Radiology and Neuroradiology, Charité, Berlin, Germany; ^4^ Emory Center for Heart Disease Prevention, Emory University School of Medicine, Atlanta, GA, United States; ^5^ Department of Global Health, Boston University School of Public Health, Boston, MA, United States; ^6^ Division of Infectious Diseases and Global Public Health, University of California, San Diego, San Diego, CA, United States

**Keywords:** google trends, infodemiology, SARS-CoV-2 vaccination, influenza vaccination, flu vaccination

## Abstract

Stagnating COVID-19 vaccination rates and vaccine hesitancy remain a threat to public health. Improved strategies for real-time tracking and estimation of population-level behavior regarding vaccinations are needed. The aim of this study was to evaluate whether online search trends for COIVD-19 and influenza mirror vaccination rates. State-level weekly fraction of online searches for top vaccination-related search terms and CDC vaccination data were obtained from June 1, 2020, to May 31, 2021. Next, trends in online search and vaccination data for COVID-19 and influenza were analyzed for visual and quantitative correlation patterns using Spearman’s rank correlation analysis. Online searches in the US for COVID-19 vaccinations increased 2.71-fold (95% CI: 1.98-3.45) in the 4 weeks after the FDA emergency authorization compared to the precedent 4 weeks. In March-April 2021, US online searches reached a plateau that was followed by a decline of 83.3% (95% CI: 31.2%-135.3%) until May 31, 2021. The timing of peaks in online searches varied across US states. Online searches were strongly correlated with vaccination rates (r=0.71, 95% CI: 0.45 - 0.87), preceding actual reported vaccination rates in 44 of 51 states. Online search trends preceded vaccination trends by a median of 3.0 weeks (95% CI: 2.0-4.0 weeks) across all states. For influenza vaccination searches, seasonal peaks in September-October between 2016-2020 were noted. Influenza search trends highly correlated with the timing of actual vaccinations for the 2019-2020 (r=0.82, 95% CI: 0.64 – 0.93) and 2020-2021 season (r=0.91, 95% CI: 0.78 – 0.97). Search trends and real-world vaccination rates are highly correlated. Temporal alignment and correlation levels were higher for influenza vaccinations; however, only online searches for COVID-19 vaccination preceded vaccination trends. These findings indicate that US online search data can potentially guide public health efforts, including policy changes and identifying geographical areas to expand vaccination campaigns.

## Introduction

The ongoing coronavirus disease 2019 (COVID-19) pandemic has challenged health care systems around the world since late 2019 (1). Broad accessibility to the genetic sequence of the severe acute respiratory syndrome coronavirus 2 (SARS-CoV-2) as a well as extensive research on coronaviruses (2) were the basis for the rapid development of a vaccine (3–5). As of December 11, 2020, US Food and Drug Authority issued an emergency use authorization for the first vaccine developed by BioNTech/Pfizer, followed by the authorization of two additional vaccines developed by Moderna and J&J/Janssen for nation-wide use. Meanwhile, several SARS-CoV-2 variants with higher transmission rates and potentially decreased susceptibility to vaccine-induced immunity, or increased disease severity emerged around the world (6, 7). Studies have shown a reduced neutralization capacity of vaccines when applied in disease variants, such as the B.1.617.2 (Delta) (8–11). Vaccine inequity and hesitancy leading to low vaccination rates may contribute to the emergence of new disease variants. Thus, the optimal distribution of vaccine shots and global availability are essential to mitigate the spread of infections and prevent COVID-19. However, vaccination rates have been reported to be stagnating in the Western Hemisphere since September 2021 and thus might endanger effective population-level control of SARS-CoV-2 infections (12–14).

Over the past decade, influenza vaccination campaigns have been shown to be an important public health measure (15). During the current COVID-19 pandemic, influenza vaccinations gained particular attention because both diseases might coincide, which might bear the potential of increased risk of simultaneous infections (16–18). Key factors for an effective vaccination campaign include supply-side factors, such as efficient and timely vaccine distribution, and demand-side factors, such as an individual willingness to be vaccinated (19–21). The extent to which online searches might represent an effective tool for predicting subsequent vaccine uptake is largely unknown.

Search data from Google, the most common search engine, can be extracted from Google Trends (GT) together with information on regional and temporal specifics of online searches. GT represents an effective tool that can mirror public health trends and might predict patient behavior in response to health-related topics (22–25). The evolving research field of ‘Infodemiology’ deals with the analyses of such online health data to identify public health trends that can guide health care professionals and policymakers in decision-making and allocation of resources (26–28).

This study compares trends in online search interest for influenza and COVID-19 vaccinations and correlates these trends with actual vaccine administration rates across the United States. The overall aim is to (1) provide a comparative overview of online searches for influenza, and COVID-19 vaccinations between December 2020 and May 2021 and (2) investigate to what extent Google search data mirror or predict actual vaccination rates. These results may be of particular interest to policymakers in the light of stagnating COVID-19 vaccination rates and the rising attention on seasonal influenza vaccination campaigns.

## Materials and Methods

### Data

Data were extracted from Google Trends as query fractions per 10 million search queries for influenza and COVID-19/SARS-CoV-2 vaccination-related terms in the US using Google Trends for Health Application Programming Interface. Weekly search data were retrieved for a 12-month period between June 1, 2020, and May 31, 2021, to explore short-term trends. This timeframe was selected to cover a period of 6 months before and after the first COVID-19 vaccine approval. Monthly search queries were extracted for the analysis of long-term trends between June 1, 2016, and May 31, 2021.

The respective terms were selected using the “top related queries” field in the Google Trends application after removing unrelated/unspecific terms (e.g. “Pfizer”, “Vaccine”) as well as duplicates. Search trends for COVID-19 vaccinations were analyzed as the average of the top 10 terms related to “vaccine covid 19” (**Supplemental Table 1**). Influenza vaccination-related terms as represented by the term “flu shot” were analyzed as weekly (average of top 10 terms) and monthly data (**Supplemental Table 2**). Online search data for all 50 US states and the District of Columbia (Washington D.C., hereafter referred to as state) were extracted, and states were labeled according to the United States Postal Service Two-Letter State Code.

Vaccination data and weekly COVID-19 case numbers for all US states were retrieved from Centers for Disease Control and Prevention (CDC) databases providing publicly available information on the administered vaccine doses in the US for both influenza (National Influenza Vaccination Dashboard) and COVID-19 (CDC COVID Tracker) (29). Administered influenza vaccinations were available as weekly doses for vaccinations in pharmacies and physician medical offices as applied US-wide, while data for US states were unavailable.

### Statistical Analysis

To compare search trends, Microsoft Excel, version 16.52, was used to compute time-averaged searches and weekly COVID-19 vaccinations. The rcompanion package in R software, version 4.1.1 (R Foundation for Statistical Computing), was applied to calculate the non-parametric Spearman’s rank correlation between weekly search rates and administered vaccine doses. Comparisons between correlation coefficients were performed using Fisher’s z-transformation and Student’s t-test. Visualization of trends in vaccinations and online searches were performed using the ggplot2 package in R. We also forecasted search interest for COVID-19 vaccination-related terms pre- and post-vaccine approval using an autoregressive integrated moving average (ARIMA) starting on November 1, 2020 accounting for the first positive trial results for a COVID-19 vaccine and the FDA authorization request. December 12, 2020, was set as cut-off as this was representative for the first COVID-19 vaccine authorization in the US on December 11, 2020. ARIMA calculation was performed using the gtrendR package in R software, version 4.1.1.

## Results

### Overall Online Search Trends for COVID-19 and Influenza Vaccinations

**Figure 1:**
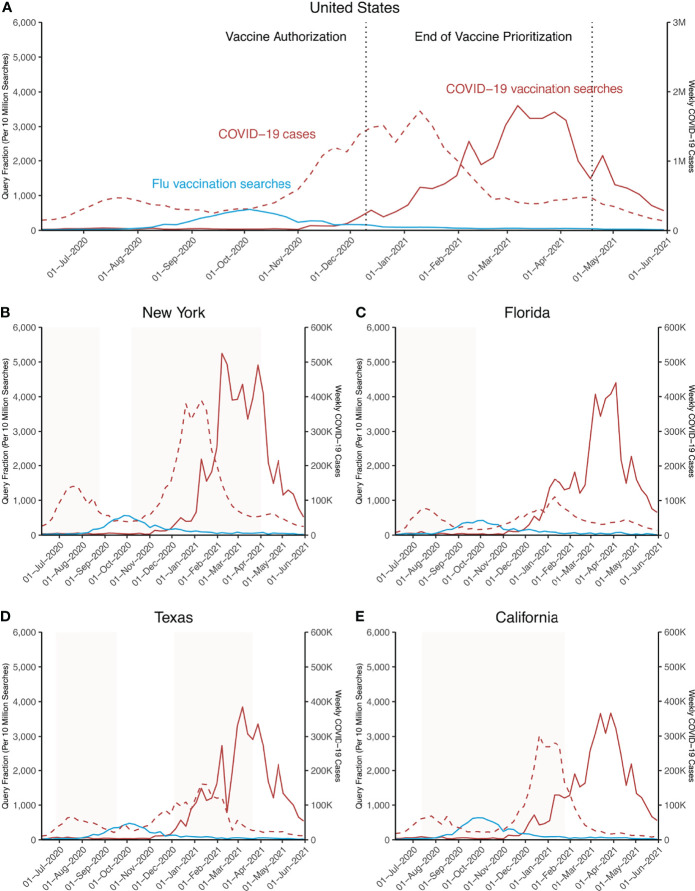
Search Trends for top 10 terms related to COVID-19/influenza vaccination in the US and selected states. Online searches from June 1, 2020, to May 31, 2021, as weekly query fraction per 10 million searches for top 10 terms related to “vaccine covid 19” (indicative of COVID-19 vaccinations) and top 10 terms related to “flu shot” (indicative of influenza vaccinations) are depicted. Data are shown for **(A)** the United Sates (US) and **(B–E)** for indicated US states. Fill color gradients (light red) represent periods of pandemic-related restrictions: **(B)** March 20, 2020 – August 25, 2020 and October 7, 2020 - April 1, 2021; **(C)** April 1, 2020 – September 25, 2020; **(D)** June 25, 2020 – September 17, 2020 and December 4, 2020 – March 10, 2021; **(E)** March 13, 2020 – January 25, 2021.

Trends in online searches, as represented by the query fraction per 10 million searches, for the top 10 terms related to COVID-19 and influenza vaccinations as well as weekly COVID-19 case numbers between June 2020 and May 2021 are shown in **Figure 1**. Searches for influenza vaccinations in the US peaked during the week of October 4, 2020. A 4.94-fold (95% CI: 4.17-5.72) increase was noted during the months of influenza vaccination recommendations when compared to precedent 2 months, July-August (**Figure 1A**). Beyond October 2020, a clear decline in online searches for influenza vaccination was observed, with a decrease of 64.5% (95% CI: 43.8%-85.1%) for the following months of November-December 2020 when compared with September – October 2020.

For searches related to COVID-19 vaccinations in the US, peaks were observed in the weeks of February 7, March 7, March 28, and April 25, 2021 (**Figure 1A**). Since the first vaccine authorization in the US, online searches increased 2.71-fold (95% CI: 1.98-3.45) for the 4 weeks after approval compared to the 4 weeks prior to vaccine approval (**Figure 1A** **).** During March 2021, online search trends reached a plateau level with highest search volumes (**Figure 1A**). Beyond the peak on March 28, 2021, online searches showed an overall decreasing trend; despite a transient peak in the week of April 25, 2021, an overall decrease of 83.3% (95% CI: 31.2%-135.3%) was observed between March 28 and May 31, 2021. Overall, weekly US COVID-19 cases peaked on January 08, 2021, while a decreasing trend could be not thereafter which was coincident with increasing vaccination rates (**Figure 1A**).

### Expected Online Searches for COVID-19 Vaccinations After Vaccine Approval

In an additional autoregressive analysis, actual online searches for selected terms representative for COVID-19 vaccination (‘vaccine covid 19’) and COVID-19 vaccine side effects (‘covid vaccine side effects’) exceeded the expected online searches after the first approval of a COVID-19 vaccine on December 11, 2021 (**Supplemental Figures 1**, **2**). Between December 12, 2020, and May 31, 2021, a difference of 205% (95% CI: 159% - 250%) for the terms ‘vaccine covid 19’ and 242% (95% CI: 209% - 278%) for ‘covid vaccine side effects’ between expected and actual online searches in the US was observed.

### State-Level Online Search Trends for COVID-19 and Influenza Vaccinations

**Figure 2:**
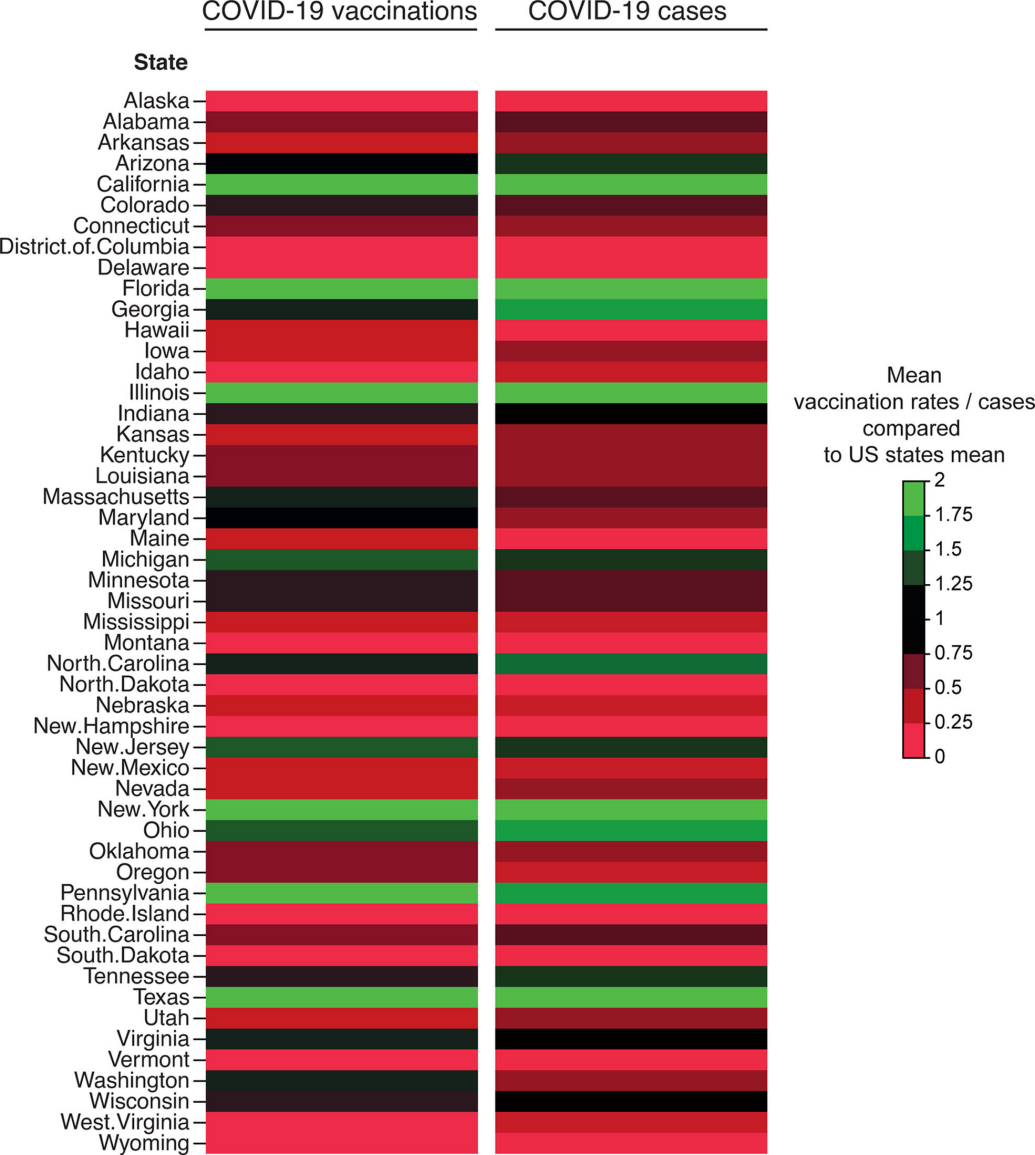
COVID-19 vaccination rates and COVID-19 cases across 51 states in relation to US trends. Means of weekly COVID-19 vaccination rates across 51 between January 10, 2021, and May 31, 2021 are shown as a fraction of the US average. For COVID-19 mean daily cases between June 1, 2020 and May 31, 2021 are depicted as a fraction of US state average. Data are shown as color-coded heatmaps by US states as indicated by the legend (green to red).

The following states showed COVID-19 vaccination rates above the US average: NY 344%, TX 299%, FL 228%, and CA 577% above the US mean between the first CDC-recorded vaccinations January 10 and May 31, 2021 (**Figure 2**). Of note, mean COVID-19 case numbers for these were as well above the US state mean (**Figure 2**). Therefore, online search trends for these states exemplary states are depicted (**Figures 1B** **–E**). Overall, search trends at the state-level for both COVID-19 and influenza vaccinations showed a similar trend compared to the US trends (**Figures 1B** **–E**). Whereas for weekly COVID-19 cases in these states, different trends could be noted (**Figures 1B** **–E**). While for CA and NY peaks in weekly infections were generally higher when compared to FL and TX; weekly peaks were reached in the week of January 10, 2021 for the US, NY, FL, TX and for CA in the week of December 20, 2020 (**Figures 1B** **–E**).

For terms related to COVID-19 vaccinations, peaks in online searches were reached in March-April 2021 with FL on April 04, TX on March 07, and CA on March 28, while searches in NY peaked earlier on February 07, 2021. Similarly, and consistent with overall US trends, online search trends for influenza vaccinations reached a maximum in late September – early October 2020 with peaks for NY, FL, and TX on October 4, 2020, and for CA on September 27, 2020 (**Figures 1B** **–E**).

### Online Searches for COVID-19 Vaccination Preceded Vaccination Rates

**Figure 3:**
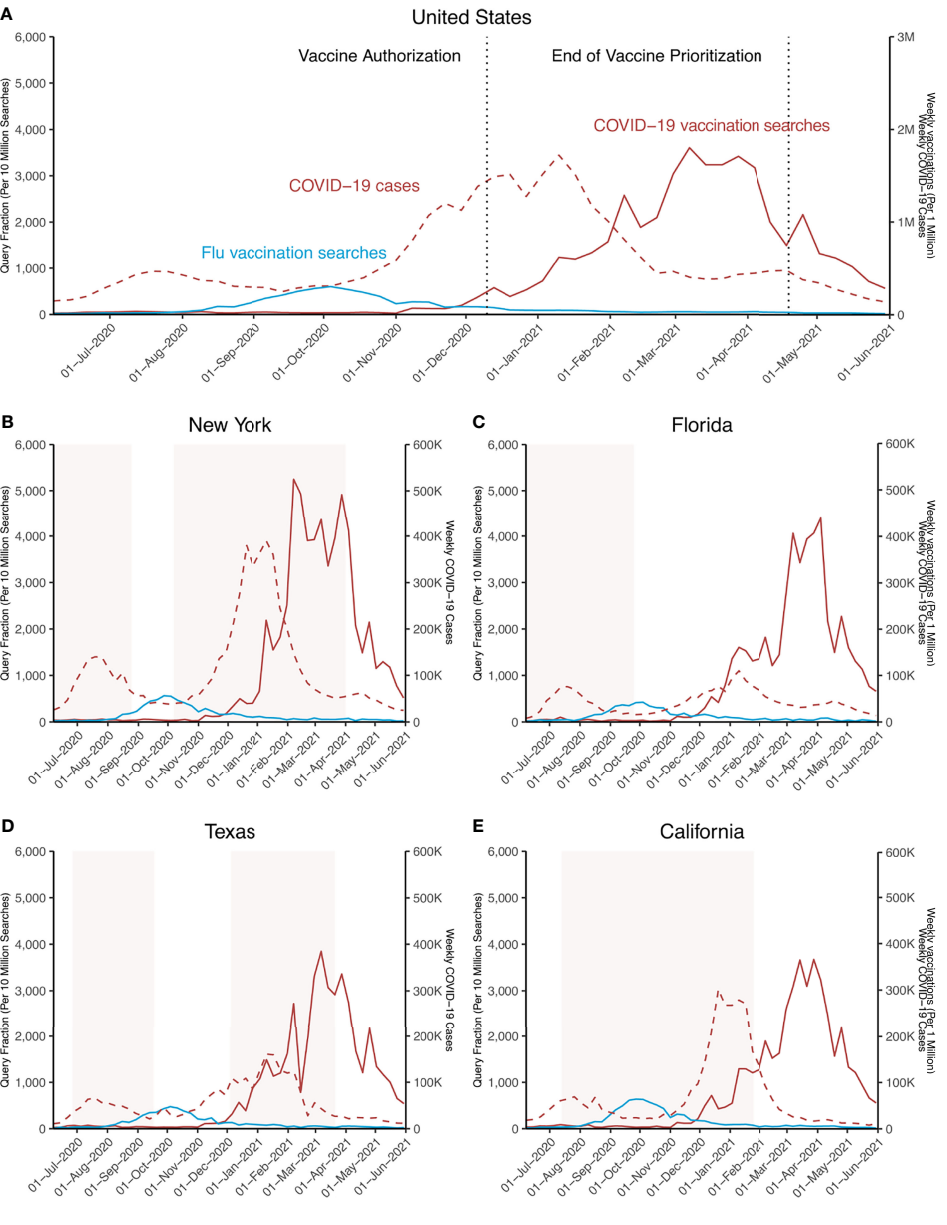
Search Trends for top 10 terms related to COVID-19 vaccination and actual vaccinations administered in the US and selected states. Online searches for the top 10 terms related to “vaccine covid 19” from June 1, 2020, to May 31, 2021, are shown. Weekly vaccinations as applied vaccinations per 1 million are depicted for **(A)** the United Sates (US) between December 20, 2020 – May 31, 2021, and **(B–E)** for indicated US states between January 10 – May 31, 2021. Fill color gradients (light red) represent periods of pandemic-related restrictions: **(B)** March 20, 2020 – August 25, 2020 and October 7, 2020 - April 1, 2021; **(C)** April 1, 2020 – September 25, 2020; **(D)** June 25, 2020 – September 17, 2020 and December 4, 2020 – March 10, 2021; **(E)** March 13, 2020 – January 25, 2021.

**Figure 4:**
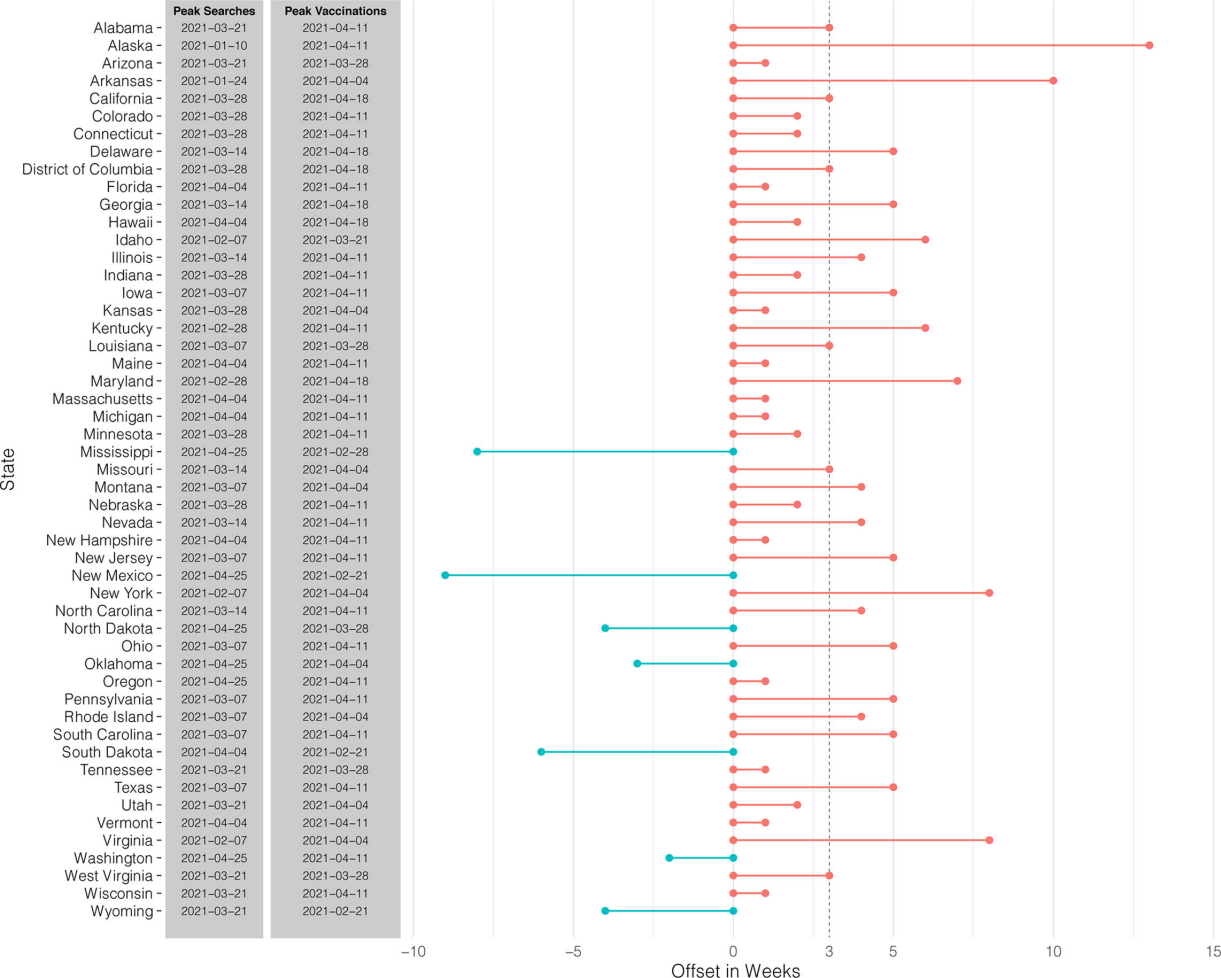
Offsets between peaks in COVID-19 vaccination online searches and vaccination rates across 51 US states. Temporal peaks of online searches for COVID-19 vaccinations between June 1, 2020 and May 31, 2021, as well as for COVID-19 vaccination rates between December 20, 2020 - May 31, 2021, across 51 US states are shown. Offsets in weeks between peaks in online searches and vaccination rates are depicted as bar graphs. The dotted line indicates the median of offsets across all shown US states.

Weekly online searches and vaccination rates per 1 million persons for COVID-19 are shown in **Figure 3**. Of note, while online searches for the US showed a clear decline beyond March 28, 2021, weekly vaccination rates increased by 14.7% (95% CI: 8.2% - 21.1%) between March 28 and April 11, 2021, with a maximum of 69,187 per million vaccinations on April 11, 2021. Beyond April 11, 2021, COVID-19 vaccination rates experienced a decline by 65.9% (95% CI: 80.0% - 51.7%) between April 11 and May 31, 2021. Generally, an offset of peaks in related online searches and vaccination rates could be noted with a median of 3.0 weeks (95% CI: 2.0-4.0 weeks) across all states (**Figures 3**, **4**). For the following exemplary states with above national average vaccination rates, peaks in vaccination rates followed peaks in online searches by an offset: NY 8 weeks, FL 1 week, CA 3 weeks, TX 5 weeks (**Figures 3B** **–E**). This trend was consistent across US states, with 44 of 51 US states showing similar trends for the temporal relationship between online searches and vaccination rates (**Figure 4**). Interestingly, for the following states, MI, NM, ND, OK, SD, WA, and WY, a negative offset can be noted indicating that peaks in vaccinations were reached before peaks online searches (**Figure 4**).

**Figure 5:**
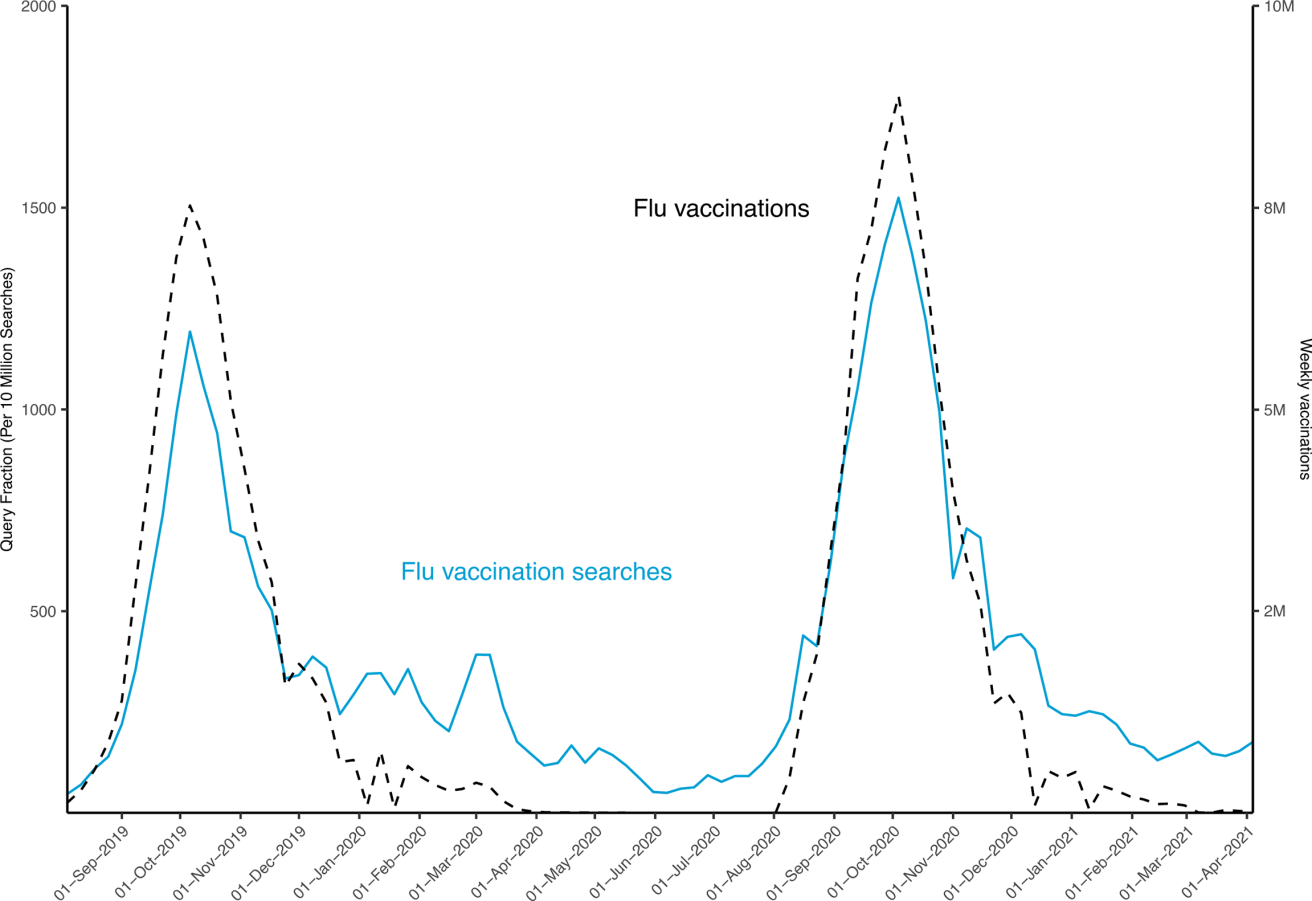
Search Trends for top 3 terms related to influenza vaccination and applied vaccinations in the US. Weekly online searches from August 4, 2019, to April 10, 2021, for the top 3 terms related to “flu shot”. Weekly vaccinations are shown for the 2019-2020 and the 2020-2021 influenza vaccination seasons: from August 4, 2019, to May 31, 2020, and August 2, 2020, to April 10, 2021.

Correlation analyses revealed a clear correlation for both COVID-19 and influenza in terms of online searches and vaccination rates (**Tables 1**, **2**). For COVID-19 vaccination-related searches and actual vaccinations, a correlation could be noted between the first CDC-recorded vaccinations for the US on December 20, 2020, and May 31, 2021 (r=0.71, 95% CI: 0.45 - 0.87) (**Table 1**). Similar but stronger levels of correlation were observed between influenza searches and vaccinations during the peaks in the 2019-2020 (August 4, 2019 - May 24, 2020) vaccination season (r=0.82, 95% CI: 0.64 – 0.93) and the 2020-2021 (August 2, 2020, and April 4, 2021) season (r=0.91, 95% CI: 0.78 – 0.97) (**Figure 5** and **Table 2**). Compared with correlations for COVD-19 vaccinations, correlations were strongest for the 2020-2021 influenza vaccination season (p=0.022), while there was no significant difference for the 2019-2020 season (p=0.317).

**Table 1:** Correlation for weekly queries per 10 million searches and COVID-19 vaccination rates.

Time	Spearman’s rho	p-value	95%-CI
Dec 20, 2020 – May 31, 2021	0.71	P<0.001	0.45 - 0.87

**Table 2:** Correlation for weekly queries per 10 million searches and influenza vaccination rates.

Time	Spearman’s rho	p-value	95%-CI
Aug 4, 2019 – May 24, 2020	0.82	P<0.001	0.64 – 0.93
Aug 2, 2020 – Apr 4, 2021	0.91	P<0.001	0.78 – 0.97

### Online Search Trends for Influenza Aligned With Vaccination Rates

Online search trends for influenza vaccinations and actual administrated vaccine doses throughout the US between 2019 and 2021 are depicted in **Figure 5**. Of note, maximum weekly online searches coincided with peaks in weekly vaccinations for the weeks of Oct 6, 2019, and Oct 4, 2020. As such, and in contrast to the findings for COVID-19 vaccinations, no offset between maximum online searches and vaccination rates for influenza vaccinations could be detected for the US (**Figure 5**). Additionally, for the 2020-2021 influenza vaccination season, peaks in weekly online searches and vaccinations increased by 28% and 18%, respectively, for the week of Oct 4, 2020, when compared to the 2019-2020 season with the week of Oct 6, 2019. Seasonal trends of online searches for influenza vaccination-related terms with peaks in the months of September-December from 2016 to 2020 were noted that consistently corresponded with the recommended months for influenza vaccinations (15) (**Supplemental Figure 3**).

## Discussion

The results of the present study show a strong correlation between online search behavior for COVID-19 and influenza vaccinations and administered vaccination doses for the US. This association was strongest for influenza vaccinations. For COVID-19 vaccinations, but not for influenza vaccinations, trends in online searches clearly preceded trends in vaccine uptake, which was consistent throughout most US states.

To our knowledge, this is the first study to evaluate the potential role of online search behavior for COVID-19 and influenza vaccinations to predict vaccination rates. The observed peaks in online searches for influenza vaccinations during September-December, which clearly aligned with vaccination rates, are in accordance with the CDC recommendations for the timing of the influenza vaccination campaigns (15, 30).

For COVID-19 vaccinations, online searches showed increases well before vaccination rates increased. One explanation for this offset between online searches and actual vaccination trends might be the potential need for information prior to an individual’s decision in favor of the vaccination. Secondly, the allocation and distribution processes, largely because of the initially limited vaccine supplies, might have contributed to the observed offset with maximum vaccination rates in April 2021. This was coincident with the removal of vaccine prioritizations making all adults eligible for vaccination across the US states (31). As such, the utilized terms included “cvs”, “vaccine near me” and “walgreens”, which might be indicative of the purpose of online searches to get information for specific vaccination sites. Additionally, the anticipation of the vaccine approval, the general availability and vaccine safety issues that were widely reported in the media might explain increased online searches, whereas vaccination trends remained behind.

In the light of stagnating vaccination rates and continuing COVID-19 vaccine hesitancy, simultaneously decreasing online search interest and vaccination rates after April 2021 might be relevant to the development and implementation of future directions in vaccination campaigns (31, 32). COVID-19 vaccine hesitancy has been reported to be a major factor for the decline of vaccination rates (33) and is reported to be largely driven by concerns about side effects (14). This has been shown to be associated with younger age, lower education status, lower household income, and the absence of health insurance (13, 14). As in those states with a negative offset (indicated by peaks in vaccinations preceding peaks in online searches) COVID-19 vaccination rates were clearly below the national average. Thus, a negative offset might be indicative for regions with delayed vaccine uptake potentially because of vaccine hesitancy. Based on the observed correlations between online searches and vaccination rates in this study, monitoring online search interest might help to forecast COVID-19 and influenza vaccination rates and indicate the potential use of trends in online searches for estimating vaccination willingness or future intentions.

Seasonal trends in influenza vaccination rates are largely a result of the recommended vaccination schedule according to CDC and guideline recommendations, which particularly indicate the months of September and October as optimal (15, 34). Online searches for terms related to influenza vaccinations revealed a clear correlation with vaccination rates. When compared to COVID-19 vaccinations, we could not observe an offset between online searches and actual vaccination rates. This might be explained by the longer experience with influenza vaccines that represent a routinely applied and effective preventive measure (35). In particular, influenza vaccinations are a common and long-term applied prevention measure with a well-known side effect profile (36). Although direct comparisons of influenza and COVID-19 vaccinations are not available, the efficacy of mRNA COVID-19 vaccines appears largely higher when compared to influenza vaccines (37–39). However, influenza vaccines are shown to reduce influenza-related hospitalizations and mortality despite a lower vaccine efficacy in older individuals (≥65 years of age) (40). Thus, the broad availability of influenza vaccines and their long-term experience with recurring vaccination campaigns is likely to be an underlying reason for stronger correlation levels and the absence of an observed offset between online searches and vaccination rates compared to COVID-19 vaccines.

### Limitations

The analyzed search terms were selected at the authors’ discretion based on the top related search terms as provided by GT to ensure overall representativity by considering multiple terms for the present analyses. During this selection process, duplicates and unrelated terms were removed to ensure relevance and specificity. There are some relevant limitations to this study: First, the use of Google searches is likely to overrepresent a younger, health-oriented population that is more likely to routinely access online information. As such, for example individuals aged ≥ 65 years might be underrepresented by online searches because of a less frequent internet usage despite generally higher vaccination rates in this age group. Second, GT data do not provide demographic or geographic information on the individual users. Therefore, subgroup analysis by demographic characteristics such as age, sex, income level could not be done. Third, the present data did not identify the purpose of vaccination-related searches. Therefore, the present search data might include online searches performed for other less related purposes, such as research or educational purposes. Ultimately, GT exclusively reports data from the Google search engine, which represents the most frequently used online search tool.

## Conclusion

We observed a strong correlation of online search volumes with vaccination rates for both COVID-19 and influenza, while for COVID-19 vaccinations, online searches precede trends in vaccinations by about 3.0 weeks (95% CI: 2.0-4.0 weeks). Thus, our findings provide support to the use of online search behavior for tracking both COVID-19 and influenza vaccination behavior and may be able to mirror COVID-19 vaccination rates. In addition, these data might complement directly gathered population-based data and provide additional guidance for decision-makers to optimize vaccination communication and vaccine distribution in vaccination campaigns. Overall, the observed correlations between online searches and actual vaccination rates for COVID-19 add further details in the predictive capability of GT for near-real-time public health analyses.

## Data Availability Statement

The original contributions presented in the study are included in the article/**Supplementary Material**. Further inquiries can be directed to the corresponding author.

## Author Contributions

PB, LH and OD participated in the conception and drafted the manuscript. AR, EB, NO, AS, SM, JA and MB revised subsequent drafts critically for important intellectual content. All authors contributed to the article and approved the submitted version.

## Funding

Author OD received support from National Institutes of Health grant T32 HL007227.

## Conflict of Interest

The authors declare that the research was conducted in the absence of any commercial or financial relationships that could be construed as a potential conflict of interest.

## Publisher’s Note

All claims expressed in this article are solely those of the authors and do not necessarily represent those of their affiliated organizations, or those of the publisher, the editors and the reviewers. Any product that may be evaluated in this article, or claim that may be made by its manufacturer, is not guaranteed or endorsed by the publisher.

## Abbreviations

CDC, Centers for Disease Control and Prevention; COVID-19, Coronavirus disease 2019; CA, California; FL, Florida; GT, Google Trends; NY, New York; SARS-CoV-2, Severe acute respiratory syndrome coronavirus 2; TX, Texas; US, United States.
