# Supplementary material for: Association of Online Search Trends With Vaccination in the United States: June 2020 Through May 2021
Source: Front Immunol. 2022 Apr 20;13:884211. doi: 10.3389/fimmu.2022.884211 (PMC9066639; doi:10.3389/fimmu.2022.884211)
Supplement: Supplementary file 1 [file DataSheet_1.pdf]

Supplementary Materials

Association of online search trends with vaccination in the United States: June 2020 through May 2021

Philipp Berning MD[1,2], Leu Huang[1,3], Alexander C. Razavi MD MPH PhD[1,4], Ellen Boakye MD MPH[1], Ngozi Osuji MD MPH[1], Andrew C. Stokes MD PhD[5], Seth S. Martin MD MHS[1], John W. Ayers MA PhD[6], Michael J. Blaha MD MPH[1], Omar Dzaye MD MPH PhD[1,3]

[1] Johns Hopkins Ciccarone Center for the Prevention of Cardiovascular Disease, Johns Hopkins University School of Medicine, Baltimore, MD, United States

[2] Department of Hematology and Oncology, University Hospital Muenster, Muenster, Germany

[3] Department of Radiology and Neuroradiology, Charité, Berlin, Germany

[4] Emory Center for Heart Disease Prevention, Emory University School of Medicine, Atlanta, GA, United States

[5] Department of Global Health, Boston University School of Public Health, Boston, MA, United States

[6] Division of Infectious Diseases and Global Public Health, University of California, San Diego, CA, United States

Supplementary Figure Legends

Supplemental Figure 1. ARIMA analysis for selected COVID-19 vaccination related terms.
Trends in actual and expected searches from November 7, 2020 to May 31, 2021 for the term 'vaccine covid 19'. Cut-off date for ARIMA analysis was December 12, 2021. The highlighted area represents differences between observed and expected search rates.

Supplemental Figure 2. ARIMA analysis for selected COVID-19 vaccination related terms.
Trends in actual and expected searches from November 7, 2020 to May 31, 2021 for the term 'covid vaccine side effects'. Cut-off date for ARIMA analysis was December 12, 2021. The highlighted area represents differences between observed and expected search rates.

Supplemental Figure 3. Long-term online search trends for terms related to influenza vaccination between 2016 and 2021.
Monthly online search volumes for the terms related to 'flu shot' as representative for influenza vaccinations, namely 'flu shot', 'flu shots' and 'flu vaccine' are shown. Data are shown as mean search fraction of these top3 terms from June 1, 2016 to May 31, 2021.

Supplemental Figure 1. ARIMA analysis for selected COVID-19 vaccination related terms.

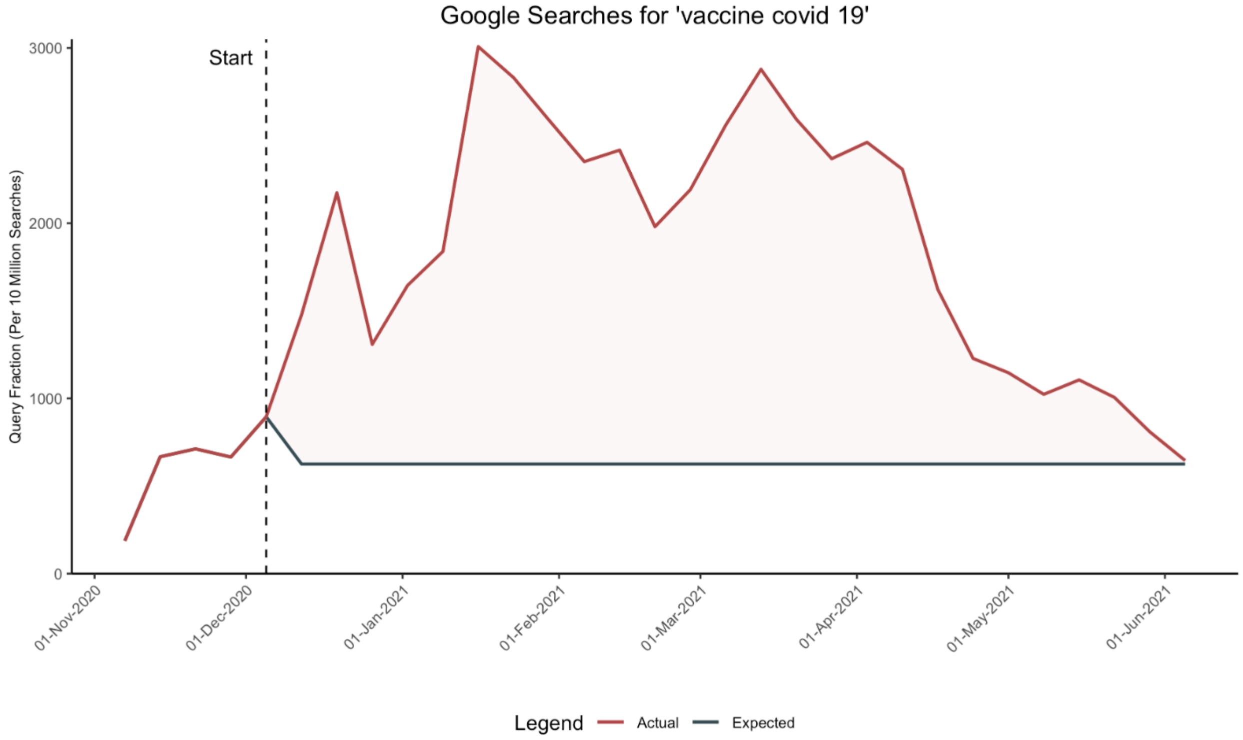

Supplemental Figure 2. ARIMA analysis for selected COVID-19 vaccination related terms.

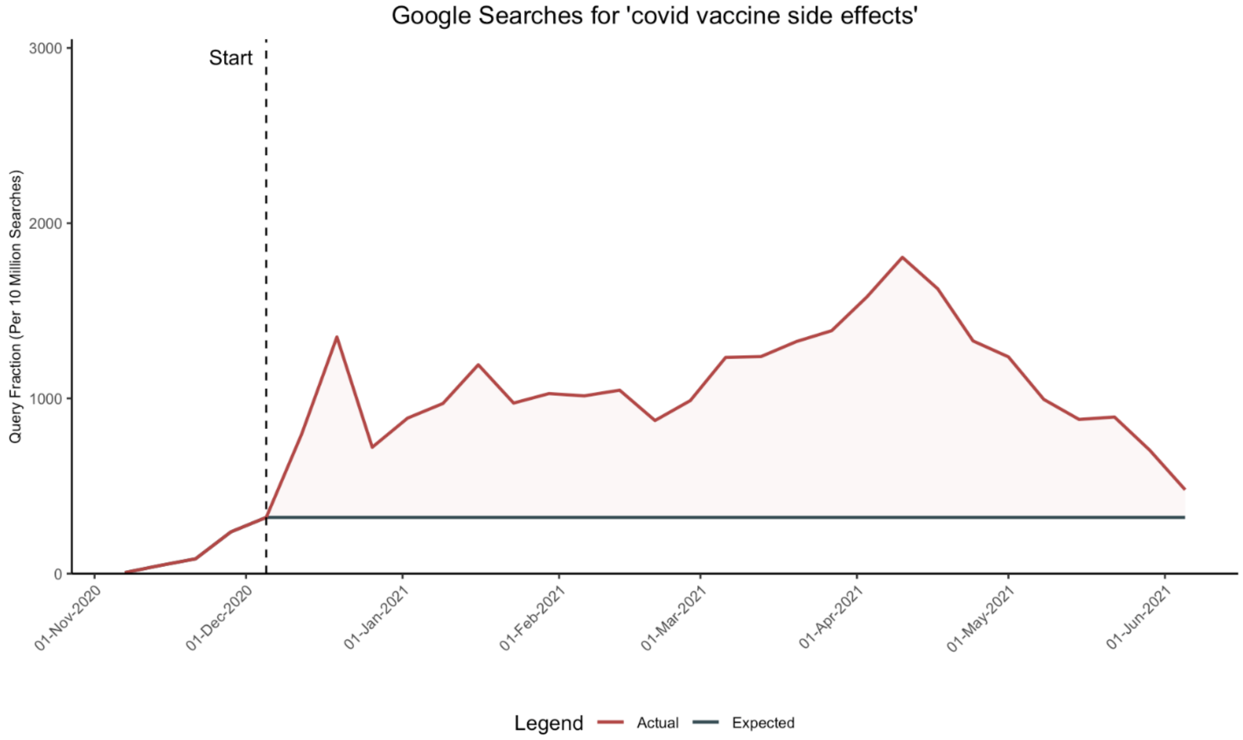

Supplemental Figure 3. Long-term online search trends for terms related to influenza vaccination between 2016 and 2021.

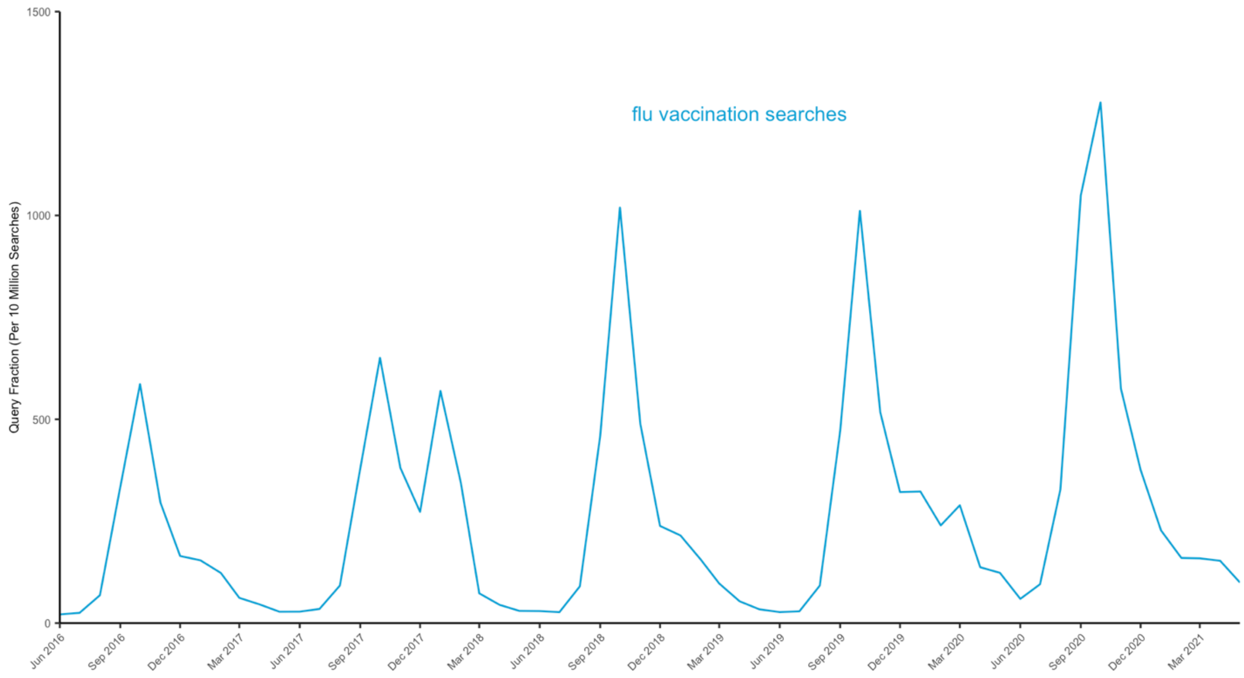

Supplemental Table 1. Top related search terms for "vaccine covid 19" in the US (June 01, 2020 – May 31, 2021).

**Search terms**
*"Vaccine covid 19"**
"Covid 19"
*"Covid vaccine cvs"**
*"Cvs vaccine"**
*"Covid vaccine near me"**
*"Covid after vaccine"**
*"Walgreens vaccine covid"**
*"Walgreens vaccine"**
*"Covid vaccine side effects"**
*"Covid vaccine appointment"**
"Pfizer"
*"Pfizer covid vaccine"**
"Pfizer vaccine"
"Covid vaccine schedule"
"Moderna vaccine"
"Moderna covid vaccine"
"Covid vaccines"
"Rite aid"
"Rite aid covid vaccine"
"Walmart vaccine covid"
"Ny covid vaccine"
"Cdc covid vaccine"
"Cdc covid"
"Florida covid vaccine"
"Nj [New Jersey] covid vaccine"

** Indicated terms were selected for further analyses and considered as 'top 10'.*

Supplemental Table 2. Top related search terms for “flu shot” in the US (June 01, 2020 – May 31, 2021).

Search terms
*“Flu shot”**
“Vaccine”
*„Flu vaccine “**
*“Flu shots”**
*“Cvs flu shot”**
*“Flu shot 2020”**
“2020 flu shot”
“Flu shot covid”
*“Flu shot side effects”**
*“Flu shot walgreens”**
*“Flu shot near me”**
“Influenza”
“Flu shots near me”
*“Flu vaccine 2020”**
“2020 flu shots”
“Free flu shots”
“Kaiser”
*“Influenza vaccine”**
“Flu symptoms”
“Free flu shot”
“Flu shots cvs”
“Flu shot symptoms”
“Kaiser flu shot”
“Flu vaccine side effects”
“Side effects of flu shot”

** Indicated terms were selected for further analyses and considered as ‘top 10’.*
